# Wash Testing of Electronic Yarn

**DOI:** 10.3390/ma13051228

**Published:** 2020-03-09

**Authors:** Dorothy Anne Hardy, Zahra Rahemtulla, Achala Satharasinghe, Arash Shahidi, Carlos Oliveira, Ioannis Anastasopoulos, Mohamad Nour Nashed, Matholo Kgatuke, Abiodun Komolafe, Russel Torah, John Tudor, Theodore Hughes-Riley, Steve Beeby, Tilak Dias

**Affiliations:** 1 The Advanced Textiles Research Group, School of Art and Design, Nottingham Trent University, Nottingham NG1 4FQ, UK; zahra.rahemtulla2018@my.ntu.ac.uk (Z.R.); achalasa@masholdings.com (A.S.); arash.shahidi@ntu.ac.uk (A.S.); jose.oliveira@ntu.ac.uk (C.O.); ianastaso@gmail.com (I.A.); m.n.nashed@gmail.com (M.N.N.); matholo.kgatuke@ntu.ac.uk (M.K.); tilak.dias@ntu.ac.uk (T.D.); 2 School of Electronics and Computer Science, University of Southampton, Highfield, Southampton SO17 1BJ, UK; a.o.komolafe@soton.ac.uk (A.K.); rnt@ecs.soton.ac.uk (R.T.); mjt@ecs.soton.ac.uk (J.T.); spb@ecs.soton.ac.uk (S.B.)

**Keywords:** electronic textiles, smart textiles, electronic yarn, wash test, tumble-dry, package die, LED, thermistor, microphone, flexible circuit

## Abstract

Electronically active yarn (E-yarn) pioneered by the Advanced Textiles Research Group of Nottingham Trent University contains a fine conductive copper wire soldered onto a package die, micro-electro-mechanical systems device or flexible circuit. The die or circuit is then held within a protective polymer packaging (micro-pod) and the ensemble is inserted into a textile sheath, forming a flexible yarn with electronic functionality such as sensing or illumination. It is vital to be able to wash E-yarns, so that the textiles into which they are incorporated can be treated as normal consumer products. The wash durability of E-yarns is summarized in this publication. Wash tests followed a modified version of BS EN ISO 6330:2012 procedure 4N. It was observed that E-yarns containing only a fine multi-strand copper wire survived 25 cycles of machine washing and line drying; and between 5 and 15 cycles of machine washing followed by tumble-drying. Four out of five temperature sensing E-yarns (crafted with thermistors) and single pairs of LEDs within E-yarns functioned correctly after 25 cycles of machine washing and line drying. E-yarns that required larger micro-pods (i.e., 4 mm diameter or 9 mm length) were less resilient to washing. Only one out of five acoustic sensing E-yarns (4 mm diameter micro-pod) operated correctly after 20 cycles of washing with either line drying or tumble-drying. Creating an E-yarn with an embedded flexible circuit populated with components also required a relatively large micro-pod (diameter 0.93 mm, length 9.23 mm). Only one embedded circuit functioned after 25 cycles of washing and line drying. The tests showed that E-yarns are suitable for inclusion in textiles that require washing, with some limitations when larger micro-pods were used. Reduction in the circuit’s size and therefore the size of the micro-pod, may increase wash resilience.

## 1. Introduction

Electronically active textiles (E-textiles) have evolved considerably in the last few decades [1,2], with one critical factor towards their successful adoption and use being the ability to wash E-textile garments. This is a challenge, as supple textile structures can often withstand machine washing but electrical circuitry mounted onto textiles will not survive, as they are not designed to withstand the repeated wetting, flexing and abrasion that occur during the washing process. 

Different levels of electronics integration will affect the durability of the E-textile when washed. One way of creating washable E-textiles is to ensure that all of the electronics can be separated from the textile structures prior to washing, then replaced. This was feasible for some early E-textiles, which could be removed from the surface of the textile structure prior to washing (first generation) [3]. First generation E-textiles also include textiles where conductive tracks have been printed onto the surface [4]; wash trials using conductive inks have shown that the durability is dependent on the physical fabric [5]. Most examples of E-textile wash durability trials in the literature have used different levels of harshness in their testing protocols: This includes using a ‘hand wash’ program on a washing machine and a small number of wash cycles (four) [6], dry cleaning [6], following the American Association of Textile Chemists and Colorists 135 1-III-B (AATCC TM135: Dimensional Changes of Fabrics after Home Laundering) standard [7], using ISO 6330:2000 (Textiles—Domestic washing and drying procedures for textile testing) for up to 50 times [8] and continuous mechanical washing for 40 h [9]. 

This work focuses on the wash durability of a new generation of E-textiles, which incorporate electronics within the yarn’s structure [1] (third generation), to create an electronic yarn (E-yarn) [10,11]. E-yarn can then be processed using conventional textile fabric machinery, without any modification, to produce E-textiles: E-yarns can be integrated as floats (laid in) during knitting or inserted as a weft during weaving (as shown for a cycling jacket, where the E-yarns were used as a weft yarn in a computerized Jacquard loom [12]). E-yarns can also be couched onto textiles using embroidery machines. As a result E-yarns can be used to position sensors or active devices in discrete locations, for example temperature sensing E-yarns can take point temperature measurements of the skin (which is not possible with some alternative methods, such as calculating temperature using the resistance of a conductive wire [13]).

The E-yarns can contain any small-scale electronic component, such as a package die, to provide sensing or illuminating functions; a small flexible circuit; or a Micro-Electro-Mechanical Systems (MEMS) device. The electronic die or circuit is soldered onto a fine multi-strand copper wire (usually with a total diameter of around 130 µm) with each electronic component enveloped by a resin micro-pod [14]. Alternatively, dies can be soldered onto a circuit etched onto copper-coated polyimide film to enable advanced electronic circuits to be included within E-yarns [15]; the entire circuit can then be encapsulated with resin. Therefore, the E-yarn technology has an unparalleled range of options for the types of electronics that can be incorporated within the yarns.

Currently only limited studies of the washability of this type of E-textile have been conducted. The wash durability of E-yarns containing LEDs [16] has been reported, stating that LED E-yarns survived 7 to 25 machine washing and tumble drying cycles, however full details were not provided. Complete wash trails were conducted for both E-yarns containing photodiodes [17] and E-yarns containing solar cells [18]. For wash testing with photodiode embedded E-yarns two sets of five E-yarns were tested; five embroidered onto the surface of a cotton T-shirt, which were machine washed and tumble-dried and five woven into a textile structure, which were machine washed and line dried. The washing procedure was based on Procedure 4N of ISO 6330:2012. It was observed that 20% of the embroidered E-yarns survived 25 washing/drying cycles and that 60% of the woven E-yarns survived 25 washing/drying cycles, with the first failures not observed until after wash 15 [17]. Solar cells embedded within E-yarns were also wash tested. Ten solar cell embedded E-yarns were woven into fabrics, with five undergoing machine washing (based on Procedure 4N of ISO 6330:2012) and line drying and five subjected to hand washing (based on the AATCC Monograph M5 for Standardization of Hand Laundering for Fabrics and Apparel) and line drying: The power output of the yarns was measured after washes. It was observed that 60% of the solar cell embedded E-yarns survived 25 domestic machine washing cycles. All of the solar E-yarns survived 25 cycles of hand washing and drying and maintained ~90% of their power output by the end of the wash tests [18]. When failures occurred, breakage of the copper wire at the interface between the micro-pod and copper wire was observed. As such it is probable that larger devices will lead to breakages earlier, as larger micro-pods will result in a greater movement, putting further fatigue on the wire. From the two studies above however this was not seen to be the case: solar cell embedded E-yarn micro-pods are larger than the photodiode embedded E-yarns, yet under comparative testing conditions both saw 40% failures after 25 machine washing and line drying cycles. In order to fully understand electronic yarn failures during washing a comparative study of a range of E-yarns, with different sizes of components, was therefore necessary. As E-yarn have now been incorporated into prototype garments [16,19,20] the necessity to conduct these wash tests had become more pressing.

This paper thoroughly evaluates the wash durability of four different types of E-yarn (six variants in total): copper wire E-yarns [11]; illuminating E-yarns [12,16], temperature sensing E-yarns [19] and acoustic sensing E-yarns [21]. This range of devices provided micro-pods with a range of sizes spanning the smallest micro-pods typically used for E-yarns (for thermistors and LEDs) to the largest (for microphones). This selection of devices, combined with the data from earlier studies also covered the majority of extant E-yarns. Copper wire E-yarn experiments were conducted to both better understand the cause of wash failures and to characterise the durability of these yarns. This information might also be useful to the community more generally, as to the knowledge of the authors wash durability of copper wires has not previously been disclosed in the literature. 

For this study, machine washing and both line drying and tumble drying methods were used in this work with the procedures being informed by ISO 6330:2012. E-yarns were tested for 25 washing and drying cycles each.

## 2. Materials and Methods 

### 2.1. Electronic Yarn Fabrication

**Figure 1:**
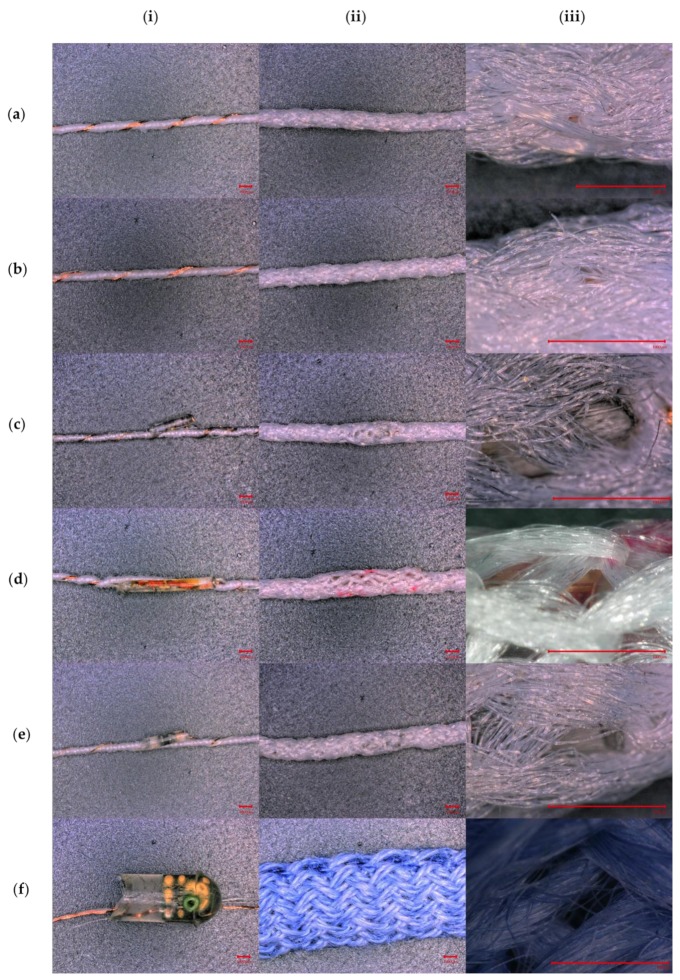
Microscope images of the E-yarns used in this work (**i**) before covering with a knit braid (**ii**) final yarn. (**iii**) High magnification image of the knit braid, showing coverage. (**a**) Conductive wire E-yarn. (**b**) Conductive wire E-yarn with a supporting yarn. (**c**) Illuminated E-yarns containing LEDs. (**d**) LEDs mounted on polyimide strip circuits embedded within the E-yarns. (**e**) Temperature sensing E-yarn. (**f**) Acoustic sensing E-yarns. Scale bar shows 1 mm.

The six variants of E-yarn explored in this work were: two types of conductive copper wire E-yarn, a temperature sensing E-yarn, an illuminated E-yarn with a light emitting diode (LED) attached to a flexible circuit, a second type of illuminating E-yarn (containing two LEDs and no flexible circuit) and an acoustic sensing E-yarn. Each type of E-yarn is shown in Figure 1, below. Figure 1 also shows a high-magnification image of the knit-braid of each E-yarn, at the micro-pod (where appropriate), showing the high level of coverage that this technique provides. E-yarns with similar designs have previously been described in the literature [11], however the exact construction methods used in this work will be described here for completeness. 

#### 2.1.1. Conductive Wire E-Yarn Fabrication

The cores of the conductive wire E-yarns were made from a 130 µm diameter, seven-strand copper wire (50 µm individual strand diameter; Knight Wire, Potters Bar, UK) which was also used to create the interconnections for all of the E-yarns described in this work. Multi-strand copper wire was used for interconnections as it is highly flexible and almost ten times more conductive than conductive yarns [22] and it is low cost. The alternative silver coated nylon yarns that can be used within E-textiles cannot be soldered to dies. Three cotton yarns (NM 30/1*2 Davidoff; Boyar Textile, Istanbul, Turkey) were twisted around the copper wire [11] using an Agteks DirecTwist 2B6 machine (Agteks, Istanbul, Turkey) at 10 m min^−1^. The copper wire was subsequently surrounded by 4 polyester packing yarns (48 f/167 dtex polyester yarns: J. H. Ashworth and Son Ltd., Hyde, UK) and inserted into a tubular warp knitted structure that was formed from 6 polyester yarns (36 f/167 dtex: J. H. Ashworth and Son Ltd., Hyde, UK) using a small-diameter circular warp-knitting machine (6 needles, 2 mm cylinder diameter; RIUS MC small-diameter warp knitting machine, RIUS, Barcelona, Spain). This resulted in a final E-yarn with a diameter of 1.5 mm. Ten conductive wire E-yarns of this design were tested. An additional batch of ten conductive wire E-yarns were also constructed with the inclusion of a Vectran™ multifilament yarn (Kuraray, Tokyo, Japan) in parallel with the copper wire, to ascertain whether this increased wash durability. The tested yarn samples were 300 mm long. 

#### 2.1.2. Temperature Sensing E-Yarn Fabrication

The general construction of temperature sensing E-yarns is well reported in the literature [19,23]. For this work temperature sensing E-yarns were fabricated using a semi-automated pilot production system (previously detailed elsewhere [11]). Negative thermal coefficient thermistors (Murata 10 kΩ 100 mW 0402 SMD NTC thermistors, Part number NCP15XH103F03RC; Murata, Kyoto, Japan) were used in this study as they were small (1.0 × 0.5 × 0.5 mm) and could be reliably soldered using the semi-automated production system. 

To create the temperature sensing E-yarns, thermistors were first soldered onto the seven strand copper wire using solder paste (lead-free, antimony-free, rosin-based solder paste, part number 7024454: Nordson EFD, Dunstable, UK) and an infrared reflow soldering process (ATN LBS-G400; ATN, Berlin, Germany). The soldered thermistors, solder-joints and a supporting yarn (Vectran™) were subsequently encapsulated within an ultra-violet curable polymer resin micro-pod (diameter = 0.94 ±0.04 mm, length = 4.2 ± 0.49 mm) using an encapsulation system (as detailed elsewhere [14]). Three of cotton yarns (NM 30/1*2 Davidoff: Boyar Textile, Istanbul, Turkey) were then twisted around the copper wire populated with micro-pods using an Agteks DirecTwist 2B6 machine (Agteks, Istanbul, Turkey). The ensemble and four polyester packing yarns were then inserted into a circular warp-knitting machine as described in Section 2.1.1 above. Six polyester yarns were used to create a sheath around the electronics, forming a final E-yarn with a diameter of 1.2 mm and length of 300 mm. Ten temperature sensing E-yarns were produced and tested in this work.

#### 2.1.3. Illuminated E-Yarn Fabrication (No Flexible Circuit)

The illuminated E-yarns were first created by soldering two LEDs (Kingbright KPHHS-1005SYCK 2.5 V Yellow LED 1005 (0402) SMD package; Kingbright, Taipei, Taiwan) in series at a minimum distance of 85.0 mm apart. As with the thermistor selection, this type of LED was chosen as it was small and could be reliably soldered. The E-yarn fabrication process was otherwise identical to the process used to create the temperature sensing E-yarns (described above). The LEDs were covered in discrete micro-pods with a diameter of 0.96 ± 0.03 mm and length of 3.51 ± 0.84 mm. The final E-yarn test samples had a diameter of 1.28 ± 0.22 mm and were 300.0 mm long. Ten illuminated E-yarns of this type were used in this work.

#### 2.1.4. Illuminated E-Yarn Containing a Flexible Circuit Fabrication

The flexible circuit used to create this type of E-yarn was supplied by the Smart Electronic Materials and Systems Research Group at the University of Southampton. Use of flexible circuits such as this could enable complex, multi-pad electronic components to be integrated into an E-yarn. This circuit was made from copper-coated polyimide (780ED Foil; GTS Flexible Materials, Ebbw Vale, UK). The foil consisted of a 25 µm thick polyimide film, 15 µm thick polyurethane based adhesive film and 18 µm thick copper sheet. The three sheets were laminated together in a roll press by the manufacturer. A 4.8 × 0.5 mm × 43 μm circuit was created on this using a standard photolithography process and subsequent wet copper etch; the process is described further in Reference [15]. An ultrathin (250 µm) 0402 imperial packaged LED (604-APG1005SECET; Kingbright Corporation, Issum, Germany) was soldered onto the circuit using a 100 µm thick stainless steel stencil and lead free solder paste (LFS-UFP, BLT Circuits, Brome, UK) at 230 °C using a hot plate for 60 s. An underfill adhesive (Loctite 4902, Henkel Adhesives, Düsseldorf, Germany) was applied between the solder pads to improve adhesion of the LED package to the polyimide substrate. 

The entire circuit, with attached seven-strand copper wire (50 µm individual strand diameter; Knight Wire, Potters Bar, UK) and multifilament Vectran™ yarn, was encased within a tubular micro-pod with a diameter of 0.93 ± 0.04 mm and a length of 9.23 ± 0.49 mm, using the resin and procedure described above and elsewhere [14]. Three cotton yarns were twisted around the entire construction. These prevented the copper wire from protruding through the outer knitted sheath. This was added by surrounding the ensemble with four packing yarns and a knitted sheath made from six polyester yarns, as described in Section 2.1.1 above.

#### 2.1.5. Acoustic Sensing E-Yarn Fabrication

Acoustic sensing E-yarns were produced using a hand-craft process by embedding MEMS microphones (3.8 mm × 1.3 mm × 3.0 mm; PMM-3738-VM1010-R, PUI Audio; Dayton, OH, USA) into E-yarns. This type of MEMS microphone was selected after testing other MEMS microphones and because this microphone had moisture and dust ingress protection [24], which was highly important for a device that was to be washed.

MEMS microphones were first soldered onto copper wire using an IR reflow station (PDR IR-E3 Rework System; PDR Design and Manufacturing Centre, Crawley, UK). As discussed in earlier work, the microphones operated without external power and only two solder joints (the voltage output terminal and one of the wake-on-sound terminals) were made. To allow for the correct operation of the microphone the resin micro-pod (diameter = 3.99 ± 0.16 mm; length 6.97 ± 0.92 mm) was formed with a small cavity to allowed for the MEMS device to detect changes in the air pressure; as with the other E-yarns the encapsulation included a supporting yarn. The final E-yarns were formed from twelve 50% merino wool and 50% Draylon^®^ yarns (Part number 3496 F71 NM 25/2; Folco, Alte Ceccato, Italy) using a small-diameter warp knitting machine (12 needles, 8 mm cylinder diameter; RIUS, Barcelona, Spain). The final E-yarns had a maximum diameter of 8.5 mm and a length of 150 mm. Longer yarns were not produced for acoustic sensing E-yarn experiments as yarns exceeding this length could not reliably be attached to the available testing apparatus. In this work ten acoustic sensing E-yarns were tested.

### 2.2. Testing the Functionality of the Electronic Yarns

The tests described in the following sections were carried out to ensure the correct functionality of the E-yarns. The tests were performed after each wash/dry cycle from washes 0–5 (except for the conductive copper wire E-yarns) and then after every five wash/dry cycles, finishing after wash 25. Each type of E-yarn required a different type of test. All tests were carried out by connecting instruments to the copper wires that protruded from the ends of the E-yarns. All microscope images were taken using a Keyence VHX-5000 microscope (Keyence, Milton Keynes, UK).

#### 2.2.1. Conductive Wire E-Yarn Testing

To ensure that the conductive wire within the E-yarns had not become damaged, resistance measurements were taken after every fifth washing/drying cycle. Resistance was measured using a multi-meter (Agilent 34410A 6½ digit; Agilent Technologies, Santa Clara, CA, USA), with the E-yarns held out straight but not under tension, during each test.

#### 2.2.2. Temperature Sensing E-Yarn Testing

Temperature sensing E-yarns were positioned on a temperature controlled plate (EchoTherm™ IC50 digital Chilling/Heating Dry Bath; Torrey Pines Scientific Inc., Carlsbad, CA, USA). Resistance measurements were taken using the multi-meter at room temperature (room temperatures varied between 19.5 and 25.7 °C during the tests that included line drying: 22.9 °C ± 1.9 °C; and from 19.8 to 24.6 °C during the tests that included tumble drying 22.5 ± 1.2 °C) and with the temperature controlled plate set to 37 °C (close to skin temperature). Air temperature was monitored using a K-type thermocouple and a data-logger (Six Channel Handheld Temperature Data Logger RDXL6SD; Omega Engineering, Inc., Manchester, UK). 

#### 2.2.3. Illuminated E-Yarn Testing

For both of the illuminated E-yarn types, experiments were conducted by powering the LEDs in the E-yarn and observing whether they illuminated or not. Power was provided using a benchtop variable power supply (IPS 3303, Iso-Tech, Corby, UK) with 4 V supplied to the illuminated E-yarn with no flexible circuit and 2 V to the illuminated E-yarn built with a flexible circuit. 

#### 2.2.4. Acoustic Sensing E-Yarn Testing

Testing of the acoustic sensing E-yarns was conducted using a bespoke acoustic chamber comprising acoustic insulation, a speaker (Bass Face SPL6M.2 800W 6.5 inch Mid-Bass Car Speaker Single; Base Face, Macclesfield, UK) and a calibrated microphone to monitor the sound level (Brüel and Kjær Type 4190 microphone with a Photon + signal analyzer; Brüel and Kjær, Nærum, Denmark). Signals recorded from the acoustic sensing E-yarn were measured through a sound card (Dynamode USB Sound Card; Dynamode UK Ltd., Watford, UK) and processed with a bespoke Python script (version 2.7; Python Software Foundation, Wilmington, DE, USA) which utilized the PyAudio [25], Matplotlib [26] and SciPy [27] modules. This produced an output amplitude given in arbitrary units (arb), which was linked to the voltage output of the microphone. The full details of the testing apparatus and conditions are detailed elsewhere in the literature [21]. 

The acoustic sensing E-yarn was deemed to be functioning correctly if, at 130 dB, it gave a response above 1665.57 arbitrary units, which was taken as the minimum output value of an acoustic sensing yarn before it was subjected to the wash (from 30 samples tested). 

### 2.3. Incorporating E-Yarns into Textiles

**Figure 2:**
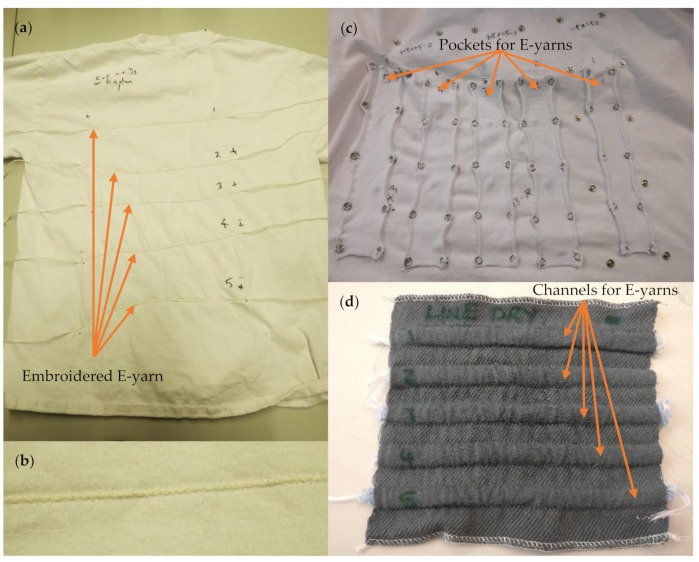
Textiles designed to hold E-yarns during wash tests: (**a**) A T-shirt with E-yarns embroidered onto the surface. (**b**) Image of an embroidered E-yarn. (**c**) Pockets on the surface of a T-shirt designed to hold E-yarns. (**d**) A woven textile with channels into which acoustic sensing E-yarns were inserted.

E-yarns were incorporated into textiles before carrying out wash tests, which would represent the way in which they would be used in real-life scenarios. All E-yarns, with the exception of acoustic sensing E-yarns, were embroidered on to the front of 100% cotton T-shirts (Pinfold, Southwell, UK) that had been prewashed at 90 °C (Figure 2a). The E-yarns were fed through a cording foot attachment on a Bernina 1000 Special sewing machine (Bernina, Steckborn, Switzerland) and secured in place with a wide zig-zag stitch (visible in Figure 2b). The ends of the E-yarns were left exposed so that crocodile clips could be attached for subsequent testing. 

The design of the acoustic testing apparatus meant that each acoustic sensing E-yarn had to be removable from the textile after washing. This was critical to ensure the correct placement and orientation of the microphone within the testing apparatus. As a result, the acoustic sensing E-yarns could not be embroidered in place in the same way as other E-yarns under test. Two methods of attachment to textiles were chosen:

1. Five acoustic sensing E-yarns were placed within a ‘pocket’ on a T-shirt (Figure 2c). The pockets were 20–25 mm across (compared to the 7 mm diameter of the acoustic sensing yarns) allowing the acoustic sensing yarns to have significant freedom of movement. The T-shirt was turned inside out before being placed in the washing machine to prevent the acoustic sensing E-yarns from getting caught on the drum of the washing machine.
2. Five acoustic-sensing yarns were inserted into woven tubes (10 mm inner diameter) within a 140 × 145 mm woven structure (Figure 2d): the structure consisted of a single cloth weave in a 4 × 4 twill, with double tubes woven in plain weave, 2/30’s combed cotton was used in both the warp and weft directions. The woven structure was placed in a wash bag, which is common practice for washing delicate textiles. This was done to prevent the acoustic sensing E-yarns from becoming caught in the drum of the washing machine, as this would place excessive mechanical forces on the yarns.

### 2.4. Wash Testing Procedures

#### 2.4.1. Water Ingress Tests for the Acoustic Sensing E-Yarns

As the cavity of the acoustic sensing E-yarn was likely to fill with water and soap during the wash tests, it was possible that the water ingress into the cavity would cause failures not seen with other E-yarn types. Before carrying out machine wash tests, it was first critical to understand if water ingress would damage the acoustic sensing E-yarns. To test this sample acoustic sensing E-yarns were placed into a beaker of tap water for 36 min: the length of the wash cycle used later and left to air dry for 24 h. Tap water was used to closely represent the type of water used in a wash cycle. After each immersion and drying cycle the acoustic sensing yarns were tested under the conditions described above (1000 Hz, 130 ± 0.56 dB). Four immersion/drying cycles were conducted.

#### 2.4.2. Machine Washing and Drying Procedure

Washing and drying were carried out in close conformance with procedure 4N in British standard BS EN ISO 6330:2012; Textiles—Domestic washing and drying procedures for textile testing [28], using a domestic front loading washing machine (Bosch Logixx 8 VarioPerfect; Bosch Classixx 8, BSH Home Appliances Ltd., Milton Keynes, UK) and 20 g of detergent (Persil Non Bio Washing Powder; Unilever UK Ltd., London, UK). The total wash load was kept at 2.00 ± 0.01 kg through use of cotton ballast (100% cotton white T-shirts). Washing was conducted at 40 °C with a 15 min washing time, 10 min of rinsing and six min of spinning (at 800 rpm).

Longer E-yarns that had been embroidered onto T-shirts tended to shrink on the first wash, puckering against the T-shirt fabric. These E-yarns were attached to T-shirts, then machine washed once and line dried prior to the start of the test, so that adjustments could be made to the embroidery, to make the E-yarns lie relatively flat on the T-shirt surface. This applied to all of the E-yarns except for the acoustic sensing E-yarns. 

Two batches of E-yarn were tested. One batch was machine washed then tumbled dried in a Bosch Classixx 8 tumble drier (Robert Bosch GmbH; Gerlingen, Germany). The drying program selected was ‘sportswear’ and ran for 1 h 47 min. The other batch of E-yarns were machine washed and then line dried indoors on an airer. The washing and drying cycles were carried out 25 times, which is the standard number of wash tests conducted for most commercial apparel, with E-yarn functionality tested after washes one to five and then after every five washes. 

### 2.5. Statistical Analysis

All error measurements presented in this paper are the standard deviation of the population unless otherwise stated. Responses from individual sensors (temperature sensing E-yarn and acoustic sensing E-yarn) have not been averaged as once an E-yarn breaks or partially breaks, the data will no longer be comparable. Therefore, the majority of the data presented is either Boolean or as a percentage of yarns that survived.

### 2.6. E-Yarn Material Properties

E-yarns are composite materials constructed from a variety of off-the-shelf materials and components. The selected materials should remain thermally stable at the relatively low temperatures experienced during washing and drying. Table 1 summarizes the key material properties of the E-yarns. The key properties include the dimensions (and micro-pod tolerances), the knitted sheath pore sizes (which gives an indication of coverage), the mechanical force needed to break the yarn and the moisture absorbency properties (which provides an indication of the micro-capillary structure within the yarn). 

All size measurements of the yarns and micro-pods were made using digital calipers (Clarke CM145 Digital Vernier Caliper; Machine Mart Ltd., Nottingham, UK). Pore size measurements were determined using a digital optical microscope (Keyence VHX-5000 Digital Microscope, Keyence (UK) Ltd., Milton Keynes, UK). Breaking force was determined with ‘dummy’ E-yarns (where the chip was not included) using a Zwick/Roell (Z2.5) tensile tester and standard ASTM D 2256/D 2256 M (Standard Test Method for Tensile Properties of Yarns by the Single-Strand Method). The tests used 320 mm long E-yarns and a grip-to-grip separation of 200 mm. Moisture absorbency was determined using 200 mm long dummy E-yarns using a Gravimetric Absorbency Testing System (M/K systems Inc., North Adams, MA, USA) following ISO 9073-12:2012 (Textiles—Test Methods for Nonwovens—Part 12: Demand Absorbency).

## 3. Results

### 3.1. Conductive Wire E-Yarn Wash Test Results

Conductive wire E-yarns were subjected to 25 washing and drying cycles. The E-yarns were considered to have failed when there was a complete lack of continuity, intermittent continuity or when the resistance exceeded 5 Ω for yarns with only copper wire, as this was well above the 0.6 to 1.1 Ω resistance measured at the start of testing (average 0.8 ± 0.18 Ω) for E-yarns that were line dried. 

**Figure 3:**
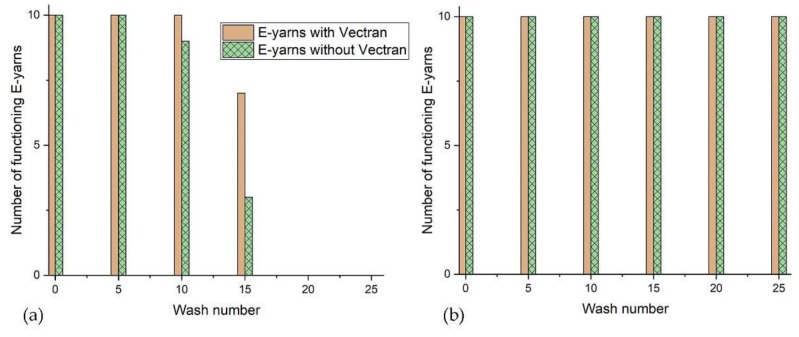
Results from wash tests for conductive wire E-yarns: (**a**) machine wash followed by tumble-drying; (**b**) machine wash followed by line drying, showing all E-yarns functioning after wash 25.

All E-yarns containing copper wire and no other electronic components still functioned after 25 cycles of machine washing and line drying (see Figure 3b). The resistance of the E-yarns containing only copper wire was between and 0.5 to 0.8 Ω after wash 25 (average 0.7 ± 0.08 Ω).

All tumble dried E-yarns had failed after wash 20. E-yarns incorporating a high tensile carrier yarn were more durable to washing and all of the E-yarns containing Vectran™ were functional after wash 10, with one E-yarn without Vectran™ having failed. Details are shown in Figure 3a.

**Figure 4:**
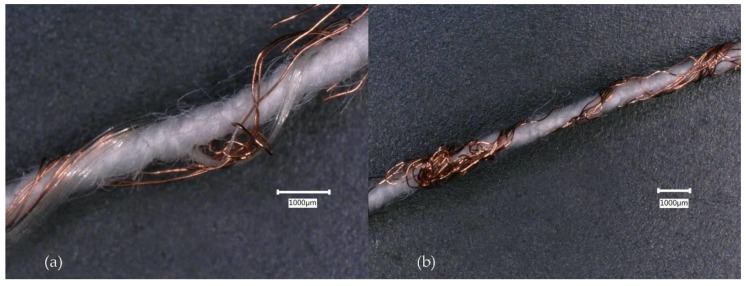
Broken strands of multi-strand copper wire removed from E-yarns after failures during wash testing with tumble-drying: (**a**) Conductive wire E-yarn with Vectran™ at 50X magnification; (**b**) Conductive wire E-yarn without Vectran™ at 30X magnification.

Electrically non-functioning E-yarns were examined by unravelling the outer, knitted sleeve. Failure was seen to be due to breaking of the individual strands of the 7-strand copper wire within each E-yarn (Figure 4). Breakages occurred at various points along the E-yarn length. The results showed that the mode of failure in the copper wire within the E-yarns were mechanical stresses. This was supported by the increased number of failures when tumble-drying was used (subjecting the yarns to an additional 1 h 47 min of mechanical stresses during each cycle).

### 3.2. Temperature Sensing E-Yarn Wash Test Results

Temperature sensing E-yarns were subjected to 25 washing and drying cycles. E-yarns were considered to have failed when no reading could be obtained from the thermistor or when the reading fluctuated continuously. 

**Figure 5:**
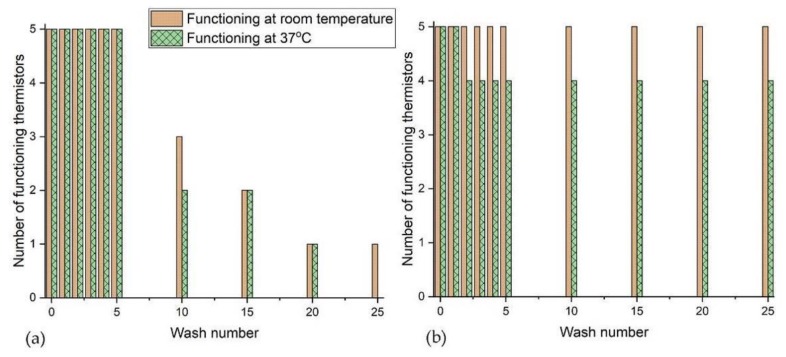
Number of functioning thermistors within E-yarns after each stage of the wash tests (**a**) with tumble-drying; (**b**) with line drying.

The bar charts in Figure 5a showed that all five temperature sensing E-yarns were still functioning after seven cycles of machine washing followed by tumble-drying. Further washing and tumble-drying caused thermistor yarn failure, with only one thermistor yarn still functioning after 18 washes. This gave only intermittent outputs after subsequent washes. The temperature sensing E-yarns that were machine washed and line dried showed a failure after the first wash (Figure 5b), however this was only observed when measurements were taken at 37 °C. The remaining E-yarns continued to function correctly after 25 washing/drying cycles.

**Figure 6:**
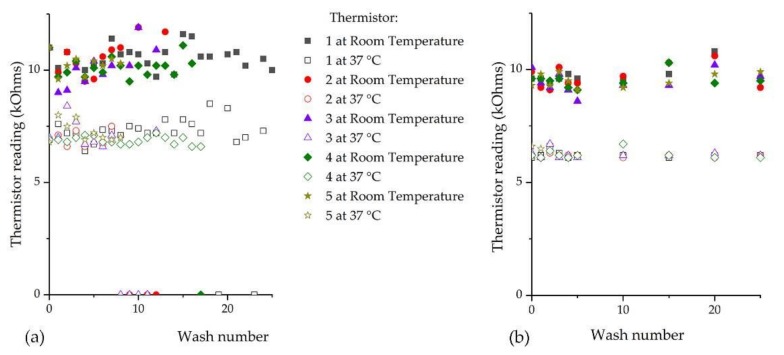
Readings from thermistors at room temperature (19.5–25.7 °C) and at 37 °C after machine washing with: (**a**) tumble-drying; (**b**) line drying. Symbols are not shown after the E-yarns physically broke.

Figure 6 shows the physical data collected from the temperature sensing E-yarns after wash testing (as raw resistance values). All five thermistors were functioning after five cycles of machine washing and tumble drying, with failures beginning to occur after this point. The spread of the readings for the temperature sensing E-yarns after machine washing/drying was 9–11.6 kΩ at room temperature and 6.4–8.5 kΩ at 37 °C; this equated to 21.2–27.8 °C and 29.3–37.2 °C respectively. Only one temperature sensing E-yarn was partially functioning after wash 25. This gave a reading at room temperature but did not function at 37 °C. 

Figure 6b shows that line dried temperature sensing E-yarns all functioned correctly after wash 25. The spread of readings between 8.6 to 10.8 kΩ at room temperature and 6.1 to 6.7 kΩ; equated to 23.0–29.0 °C and 35.9–38.5 °C respectively. This spread in results was significantly less than those observed for the temperature sensing E-yarns that were machine washed and tumble dried, as shown in Figure 6a.

**Figure 7:**
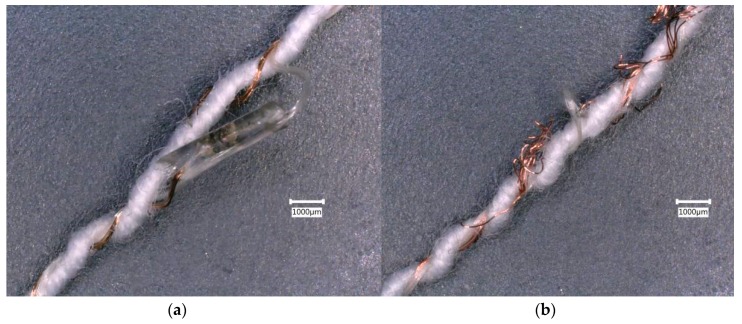
Thermistor yarns at 30X magnification: (**a**) Copper wire detached from the right side of the micro-pod; (**b**) Frayed wire greater than 50 mm away from a micro-pod.

Examination of broken thermistor yarns after wash tests showed copper wire breaking away from the edge of the micro-pod (Figure 7a), in addition to breakage of the copper wire strands at points along the entire length of the E-yarn (Figure 7b). 

### 3.3. Illuniated E-Yarn Wash Test Results

**Figure 8:**
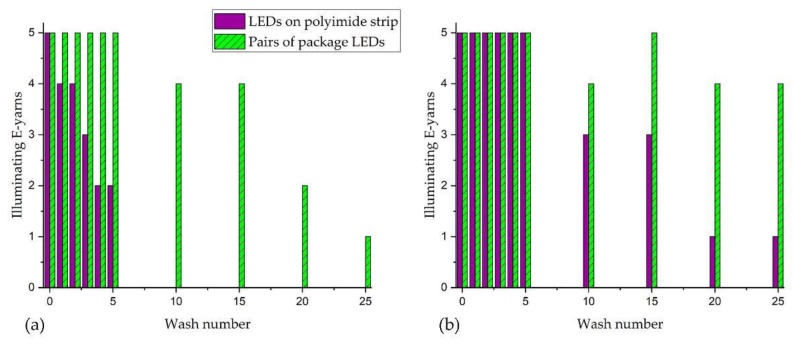
Illuminating E-yarns functioning after washing with: (**a**) tumble-drying; (**b**) line drying.

The results of the wash tests were compared for the pairs of LED package dies; and for single LEDs mounted on polyimide strip circuits embedded within the E-yarns. Figure 8 shows that machine washing followed by tumble-drying led to a steady decrease in the number of functioning LEDs for both types of E-yarn. The E-yarns containing pairs of LED package dies were more durable under these conditions, with one out of five E-yarns still functioning after 25 washes. Two of the E-yarns constructed using LEDs on polyimide strip still functioned after wash five and none after wash 10. 

Machine washing followed by line drying had a less detrimental effect on both types of E-yarn. All of the E-yarns still illuminated after five washes. Four out of five of the E-yarns constructed with pairs of package dies were still functioning after wash 25. Of these, one pair of LEDs within an E-yarn illuminated intermittently, then did not function after wash 20. Three out of the five E-yarns made using a polyimide strip circuit were still functioning after ten washes and one continued to function after 25 washes. 

**Figure 9:**
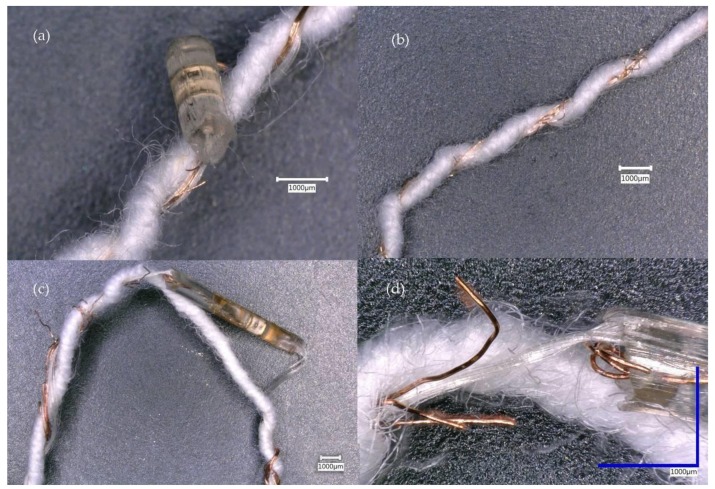
The interiors of illuminated E-yarns containing LEDs after failure during wash tests: (**a**) Copper wire severed from a micro-pod surrounding a package LED at 50X magnification; (**b**) Frayed copper wire at more than 50 mm from a package LED; (**c**) Copper wire severed from an encapsulated polyimide strip at 20X magnification; (**d**) A detailed view of (**c**) at 100X magnification.

Figure 9 shows the interiors of the illuminating E-yarns after failure during the wash tests. The copper wire had severed from the end of a micro-pod that surrounds polyimide strip with an attached LED (Figure 9c). Analysis of additional yarns showed that breakages in the copper wire occurred at or near the micro-pod interface but that additional breakage of wire strands occurred at other points along the length of the copper wire. 

### 3.4. Acoustic Sensing E-Yarn Wash Test Results

#### 3.4.1. Acoustic Sensing E-Yarn Immersion Tests

**Figure 10:**
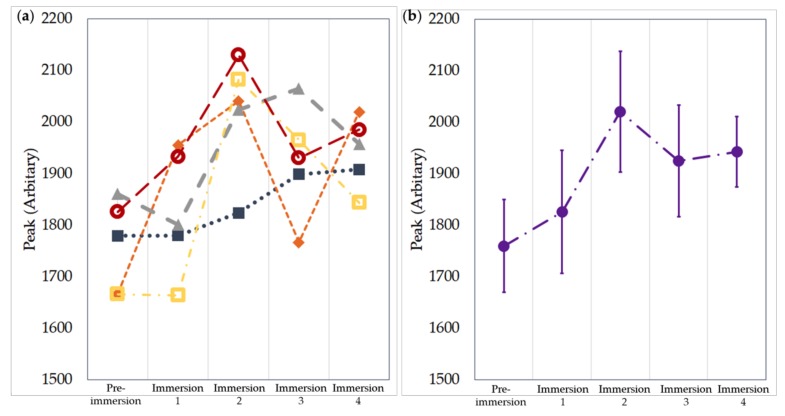
Microphone response after four immersions in water for five acoustic sensing E-yarns. (**a**) Data from individual E-yarns: yarn 1 (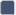), yarn 2 (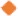), yarn 3 (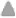), yarn 4 (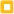), yarn 5 (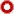). Dotted lines are included as a guide for the eye only. (**b**) Averaged data. The error bars are given by the standard deviation.

Acoustic sensing E-yarns were first tested to see if water ingress into the cavity in the encapsulation of the microphone affected the microphone response. Figure 10 shows that there was no significant reduction in the signal intensity recorded after each immersion. The VM1010-R had IPX7 water resistance, meaning that in it was capable of immersion in up to 1 m of water, which was supported by these results.

#### 3.4.2. Acoustic Sensing E-Yarn Machine Washing Tests

**Figure 11:**
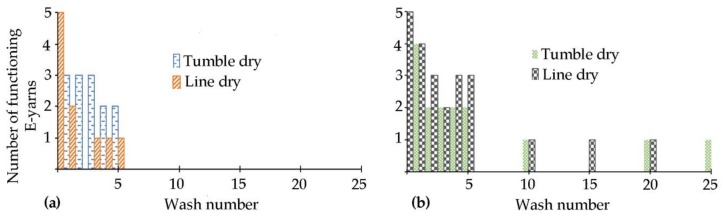
The number of working acoustic sensing E-yarns measured after wash 1, 2, 3, 4, 5, 10, 15, 20 and 25 for: (**a**) acoustic sensing E-yarns within pockets attached to T-shirts; (**b**) acoustic sensing E-yarns within channels in a woven fabric.

Figure 11a shows that by wash ten all of the acoustic sensing E-yarns washed within pockets on the T-shirts had broken. Figure 11b shows that one of each of the yarns from the woven fabric samples (one tumble-dried and one line dried) were still working. An important note is that from wash ten to 25, for the woven fabric samples, it was not always the same acoustic sensing E-yarn that was working: This was due to the variation in the peak values given for each wash. A threshold of 1665.57 arb was defined as the working value, as 1665.57 arb was the minimum output value of an acoustic sensing yarn before it was put into the wash (from 30 samples)

**Figure 12:**
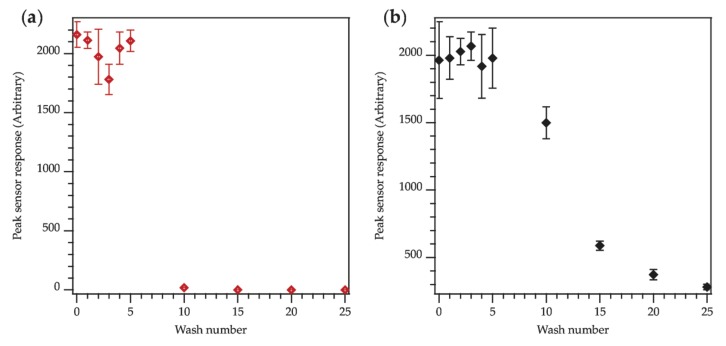
Two example datasets showing the sensor response of the acoustic sensing E-yarns over 25 washes: (**a**) The acoustic sensing E-yarn suddenly stopped working. (**b**) The performance of the acoustic sensing E-yarn degraded with multiple washes.

By recording the sensor output of the acoustic sensing E-yarns after wash tests it was observed that over multiple washing-drying cycles some acoustic sensing E-yarns suddenly stopped working, while others saw a deterioration in performance. For clarity Figure 12a shows an example where an acoustic sensing E-yarn suddenly stopped working. Figure 12b shows an acoustic sensing E-yarn where the sensor output has degraded over multiple wash cycles, which indicates damage or degradation of either the microphone or the copper wire interconnection used to collect the signal. Datasets showing the performance of the other acoustic sensing E-yarns as a function of washes are given in Supplementary Information for completeness.

#### 3.4.3. Analysis of the Acoustic Sensing E-Yarns after Machine Wash Tests

Upon the completion of the wash trials, the acoustic sensing E-yarns were dissected, and the failures were analyzed. Analysis of the E-yarns using microscopy showed that for all of the acoustic sensing E-yarns, failures were due to the copper wire interconnects becoming broken; often at the wire-micro-pod interface. No breakages inside the micro-pod were observed. 

For partially working acoustic sensing E-yarns, significant damage to the copper wires was observed. Additional tests were conducted to determine the resistance of the copper wire (for the signal-output wire). The resistance values measured varied significantly depending on the position in which the wire was held. For example, if the wire was held out straight it would give a different result to if it was allowed to bend, which most wires naturally did after washing (this was not the case for undamaged wire). These results further supported the theory that the partially working acoustic sensing yarns had damaged copper wires. Damage to the copper wires, including strand breakages, would reduce the recorded signal.

**Figure 13:**
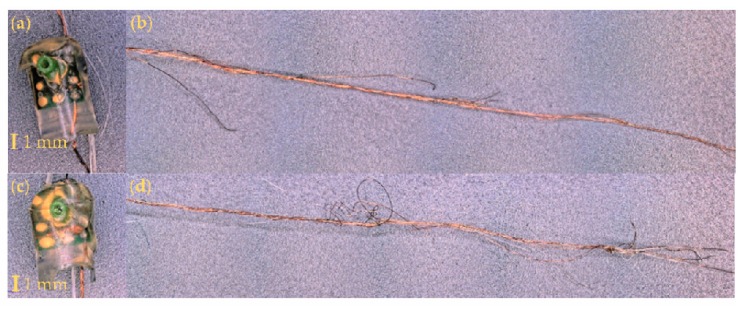
Microscope images of two acoustic sensing E-yarns that were washed within a woven structure and tumble dried. The images to the right show a section of the copper wire used to output signal: (**a**) Yarn A encapsulated microphone; (**b**) the copper wire interconnect from Yarn A; (**c**) Yarn B encapsulated microphone; (**d**) the copper wire interconnect from Yarn B.

Figure 13 shows (dissected) acoustic sensing Yarns A and B, which were from the woven fabric tumble-dry batch of wash tests. Yarn A was the only acoustic sensing E-yarn still working after wash 25, with a peak of 2079 arb and Yarn B was partially working after wash 25, with a very low peak value of 32 arb (this was the lowest peak value from the E-yarns). As seen by the microscope images, the wire of Yarn B appeared to have sustained more damage, with the copper wire showing more broken filaments; this may account for the difference in the peak values given. Given the breakages in the strands of the copper wire at different locations along the copper wire, it was not possible to quantify the damage effectively using resistance measurements. 

**Figure 14:**
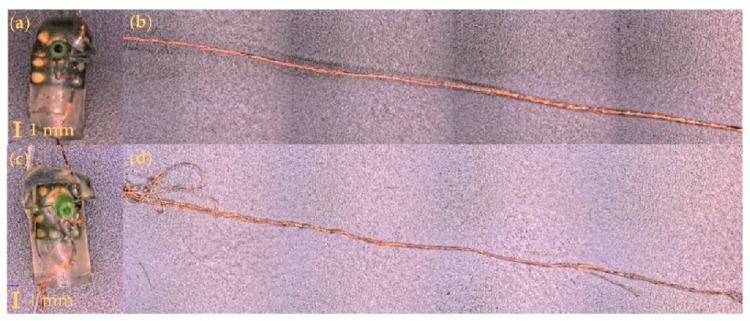
Microscope images of encapsulated microphones and the signal output wire of acoustic sensing yarns washed within a woven structure. Yarn C was line dried and had a final peak value of 1349 arb. Yarn D was tumble dried with a final peak value of 1338 arb. (**a**) Yarn C encapsulated microphone; (**b**) the copper wire interconnect from Yarn C; (**c**) Yarn D encapsulated microphone; (**d**) the copper wire interconnect from Yarn D.

Additional examples of partially working acoustic sensing E-yarns are shown in Figure 14. Yarn C and Yarn D were both washed within a woven structure with Yarn C being line dried (Figure 14a,b) and Yarn D tumble dried (Figure 14c,d). Although the sensor response was similar for both acoustic sensing yarns (1349 arb for Yarn C and 1338 arb for Yarn D) after wash 25, the images in Figure 14 showed that the wire of Yarn D appeared to be more damaged than Yarn C. The was due to the tumble-drying process putting additional mechanical strain on the acoustic sensing E-yarns when compared to line drying. 

## 4. Discussion

The key results from the wash tests have been tabulated in Table 2 below, showing the survival rates of each type of E-yarn after 25 washing and drying cycles and the cycle where the first yarn broke (to the nearest five cycles). The data only shows results where the E-yarns were attached to T-shirts. For completeness literature values for photodiode embedded E-yarns have been included (from [17]).

**Figure 15:**
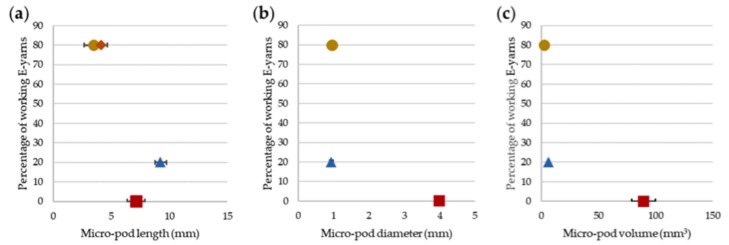
Percentage E-yarns functioning after 25 machine washing and line drying cycles as a function of: (**a**) Micro-pod length. (**b**) Micro-pod diameter. (**c**) Micro-pod volume. Illuminated E-yarns containing LEDs (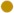), LEDs mounted on polyimide strip circuits embedded within the E-yarns (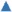), Temperature sensing E-yarn (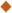; obscured in parts b and c), Acoustic sensing E-yarns (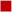).

The test results indicated that the properties of the copper wire in the E-yarns were key to wash durability. This indicated that further work was required to find a wire with suitable wash durability and conductivity. The size of the micro-pods within the E-yarns also appeared to be a factor in failure of E-yarns as a result of washing. This theory was examined further by plotting micro-pod length and volume against the number of E-yarns of each type functioning after 25 washes (Figure 15). This showed that larger micro-pods were less able to survive the rigors of washing. Figure 15a shows that the length of the micro-pod was a factor in E-yarn failure, with the microphones within micro-pods being most likely to fail, although the longer, thinner micro-pods encasing circuitry on polyimide strips were slightly less likely to fail, as one was still functioning after 25 washes. There was a clearer correlation between increased micro-pod diameter and volume and failure of the E-yarns during washing. The largest micro-pods, used for the acoustic sensing E-yarns, failed earliest, as shown in Figure 15b,c. Further miniaturization of circuitry could therefore assist in creation of E-yarns that are more durable when washing. An alternative encapsulation method developed by the authors has been used, as discussed in Reference [29]. This method was ideally suited to the polyimide strip process and shown to increase the washing robustness to 45 washes. However, this technique required creation of specific, bespoke molds for each circuit and did not lend itself as easily to mass production at this stage compared to the encapsulation process utilized in this current paper. 

## 5. Conclusions

This work demonstrated that E-yarn could be washed successfully. All twenty conductive wire E-yarns, including no additional soldered electronic components, were functional after 25 cycles of machine washing and line drying. The additional mechanical and heat stresses due to the tumble-drying process caused all of the E-yarn to fail before the 25th wash/drying cycle. The inclusion of an additional high-tensile strength yarn (Vectran™) assisted in preventing failure in the conductive wire E-yarns and seven E-yarns containing Vectran™ were still functioning after 15 cycles of washing and tumble-drying, as opposed to three of the E-yarns constructed without Vectran™. 

Temperature sensing E-yarns were also shown to be washable. Four out of five temperature sensing E-yarns continued to function correctly after 25 machine wash-line drying cycles. Only one of five temperature sensing E-yarns functioned correctly after 25 machine wash-tumble drying cycles. Tumble-drying led to erroneous temperature readings from some of the E-yarns.

Illuminating E-yarns all functioned after five machine wash and line drying cycles, with four out of five illuminating E-yarns constructed using package die LEDs surviving 25 washing/drying cycles. The illuminating E-yarns created using a flexible circuit board had a much higher failure rate, with only one out of five still functioning after 25 washing/drying cycles. The longer length of the micro-pod needed for the E-yarn containing a flexible circuit board (9.2 mm as opposed to 3.5 mm for micro-pods surrounding package LEDs) could have led to an increased strain on the copper wire at the points where it emerged from the micro-pods, causing breakage of the wire strands. Reducing the size of the circuitry sizes, together with the use of a more robust conductive wire, could increase wash resilience. Wash resistance was significantly poorer when machine washing and machine drying was used, with only one illuminating E-yarn (constructed with two package die LEDs) surviving 25 washing/drying cycles.

The acoustic sensing E-yarns had a poorer wash resilience compared to other E-yarn designs. As with the other E-yarns the failures were due to the copper wire breaking at the points where the wire emerged from the encapsulation. The larger size of the micro-pods, encasing microphones measuring (diameter = 3.99 ± 0.16 mm; length 6.97 ± 0.92 mm) may have led to earlier failure than for thermistors or packaged LEDs within E-yarns.

This study has demonstrated the durability of a variety of E-yarns to machine washing. The ability to wash E-yarns is vital to their ongoing usefulness to the E-textile industry for wearable applications, where washing is essential and machine washing desirable. 

## Figures and Tables

**Table 1:** Material properties of the E-yarns used in this study.

Type of E-Yarn	Final Yarn Thickness (mm)	Micro-Pod Length (mm)	Micro-pod Diameter (mm)	Micro-pod Volume (mm^3^)	Pore Size of the Knitted Sheath (mm^2^)	Breaking Force (N)	Absorbed Weight of Water (g)	Time to Absorb (seconds)	Absorbency Weight Capacity
Conductive wire E-yarn	1.5	N/A	N/A	N/A	0.01	92.76 ± 5.43	0.4232	21.142	2.828
Conductive wire E-yarn with a supporting yarn	1.5	N/A	N/A	N/A	0.032	88.60 ± 10.95	0.454	21.95	3.00
Illuminated E-yarns containing LEDs	1.3	3.51 ± 0.84 (2.83–5.87)	0.96 ± 0.02 (0.92–1.00)	2.53 ± 0.69 (2.18–4.52)	0.172	88.86 ± 7.72	0.466	23.77	2.94
LEDs mounted on polyimide strip circuits embedded within the E-yarns		9.24 ± 0.49 (8.41–9.88)	0.93 ± 0.04 (0.86–0.99)	6.30 ± 0.57 (5.48–7.15)	0.078	88.70 ± 6.05	0.464	22.4	2.84
Temperature sensing E-yarn	1.2	4.15 ±0.49 (3.91–4.45)	0.94 ± 0.04 (0.91–0.97)	2.89 ± 0.57 (2.63–3.29)	0.125	87.03 ± 16.47	0.432	20.65	2.84
Acoustic sensing E-yarns	8.5	7.13 ± 0.78 (6.29–8.51)	4.00 ± 0.06 (3.94–4.11)	89.47 ± 10.26 (76.69–105.87)	0.13	231.22 ± 23.60	0.081	11.85	0.067

**Table 2:** Summary of key E-yarn wash test results.

Type of E-Yarn	Line Drying		Tumble Drying	
	Percentage of E-Yarns Surviving after 25 Washing/Drying Cycles	Cycle Up to Which All E-Yarns Still Functioned	Percentage of E-Yarns Surviving after 25 Washing/Drying Cycles	Cycle Up to Which All E-Yarns still Functioned
Conductive wire E-yarn	100	N/A	0	5
Conductive wire E-yarn with a supporting yarn	100	N/A	0	10
Illuminated E-yarns containing LEDs	80	5	20	5
LEDs mounted on polyimide strip circuits embedded within the E-yarns	20	5	0	0
Temperature sensing E-yarn	80	0	0	5
Acoustic sensing E-yarns	0	0	0	0
Photodiode embedded E-yarn [17]	N/A	N/A	20	5

## Supplementary Materials

The following are available online at https://www.mdpi.com/1996-1944/13/5/1228/s1, Figure S1. Measurements from acoustic sensing E-yarns machine-washed and tumble dried within pockets attached to a T-shirt (a) An E-yarn that failed after 4 washes. (b) An E-yarn that stopped functioning after the first wash. Figure S2. Acoustic E-yarns machine washed, and tumble dried within channels in a woven textile. Each image shows acoustic measurements from the E-yarns after machine washing and tumble drying. (d) Shows results from the only E-yarn that was functioning after 25 washes. Figure S3. Acoustic E-yarns machine washed, and line dried in pockets attached to a T-shirt. Figure S4. Acoustic E-yarns machine washed, and line dried within channels in a woven textile. Results (b), (c) and (d) show partial functionality retained after 25 washes.

## References

[1] Hughes-Riley T., Dias T., Cork C. (2018). A Historical Review of the Development of Electronic Textiles. Fibers.

[2] Scott R.A. (1988). The technology of electrically heated clothing. Ergonomics.

[3] Köhler A.R., Hilty L.M., Bakker C. (2011). Prospective Impacts of Electronic Textiles on Recycling and Disposal. J. Ind. Ecol..

[4] Yang K., Torah R., Wei Y., Beeby S., Tudor J. (2013). Waterproof and durable screen printed silver conductive tracks on textiles. Text. Res. J..

[5] Shahariar H., Kim I., Soewardiman H., Jur J.S. (2019). Inkjet Printing of Reactive Silver Ink on Textiles. ACS Appl. Mater. Interfaces.

[6] Ryan J.D., Mengistie D.A., Gabrielsson R., Lund A., Müller C. (2017). Machine-Washable PEDOT: PSS Dyed Silk Yarns for Electronic Textiles. ACS Appl. Mater. Interfaces.

[7] Jin H., Matsuhisa N., Lee S., Abbas M., Yokota T., Someya T. (2017). Enhancing the Performance of Stretchable Conductors for E-Textiles by Controlled Ink Permeation. Adv. Mater..

[8] Vervust T., Buyle G., Bossuyt F., Vanfleteren J. (2012). Integration of stretchable and washable electronic modules for smart textile applications. J. Text. Inst..

[9] Du D., Tang Z., Ouyang J. (2018). Highly washable e-textile prepared by ultrasonic nanosoldering of carbon nanotubes onto polymer fibers. J. Mater. Chem. C.

[10] Dias T.K., Rathnayake A. (2017). Electronically Functional Yarns. Patent.

[11] Hardy D.A., Anastasopoulos I., Nashed M.-N., Oliveira C., Hughes-Riley T., Komolafe A., Tudor J., Torah R., Beeby S., Dias T. (2019). Automated insertion of package dies onto wire and into a textile yarn sheath. Microsyst. Technol..

[12] Hardy D.A., Townsend K., Kgatuke M., Salter E., Downes T., Harrigan K., Allcock S., Dias T. (2019). Light My Elbows: A Cycling Jacket Incorporating Electronic Yarn. Textile Intersections Conference Proceedings.

[13] National Physical Laboratory (2016). Introduction to Temperature Measurement. https://www.npl.co.uk/special-pages/guides/gpg125_intro2tempmeasure.

[14] Nashed M.-N., Hardy D., Hughes-Riley T., Dias T. (2019). A Novel Method for Embedding Semiconductor Dies within Textile Yarn to Create Electronic Textiles. Fibers.

[15] Komolafe A., Torah R., Wei Y., Nunes-Matos H., Li M., Hardy D., Dias T., Tudor M., Beeby S. (2019). Integrating Flexible Filament Circuits for E-Textile Applications. Adv. Mater. Technol..

[16] Hardy D., Moneta A., Sakalyte V., Connolly L., Shahidi A., Hughes-Riley T. (2018). Engineering a Costume for Performance Using Illuminated LED-Yarns. Fibers.

[17] Satharasinghe A., Hughes-Riley T., Dias T. (2018). Photodiodes embedded within electronic textiles. Sci. Rep..

[18] Satharasinghe A., Hughes-Riley T., Dias T. (2019). An investigation of a wash-durable solar energy harvesting textile. Prog. Photovolt. Res. Appl..

[19] Hughes-Riley T., Lugoda P., Dias T., Trabi C., Morris R. (2017). A Study of Thermistor Performance within a Textile Structure. Sensors.

[20] Lugoda P., Hughes-Riley T., Oliveira C., Morris R., Dias T. (2018). Developing Novel Temperature Sensing Garments for Health Monitoring Applications. Fibers.

[21] Hughes-Riley T., Dias T. (2018). Developing an Acoustic Sensing Yarn for Health Surveillance in a Military Setting. Sensors.

[22] Bahadir S.K., Kalaoğlu F., Jevšnik S. (2015). The use of hot air welding technologies for manufacturing e-textile trasmission lines. Fibers Polym..

[23] Lugoda P., Hughes-Riley T., Morris R., Dias T. (2018). A Wearable Textile Thermograph. Sensors.

[24] News—PUI Audio|A Projects Unlimited Company located in Dayton, Ohio. http://www.puiaudio.com/news.aspx.

[25] PyAudio PortAudio v19 Python Bindings. http://people.csail.mit.edu/hubert/pyaudio/.

[26] Hunter J.D. (2007). Matplotlib: A 2D graphics environment. Comput. Sci. Eng..

[27] SciPy Open Source Scientific Tools for Python. http://www.scipy.org/.

[28] British Standards Institution (2012). BS EN ISO 6330:2012—Textiles. Domestic Washing and Drying Procedures for Textile Testing.

[29] Li M., Tudor J., Liu J., Torah R., Komolafe A., Beeby S. (2019). Novel Electronic Packaging Method for Functional Electronic Textiles. IEEE Trans. Components Packag. Manuf. Technol..

