# Supplementary material for: Wash Testing of Electronic Yarn
Source: Materials (Basel). 2020 Mar 9;13(5):1228. doi: 10.3390/ma13051228 (PMC7085099; doi:10.3390/ma13051228)
Supplement: Supplementary file 1 [file materials-13-01228-s001.pdf]

materials

Supplementary Materials: Wash Testing of Electronic Yarn: Acoustic Sensing Yarn Results

Dorothy Anne Hardy, Zahra Rahemtulla, Achala Satharasinghe, Arash Shahidi, Carlos Oliveira, Ioannis Anastasopoulos, Mohamad Nour Nashed, Matholo Kgatuke, Abiodun Komolafe, Russel Torah, John Tudor, Theodore Hughes-Riley, Steve Beeby , and Tilak Dias

S1. Measurements from Acoustic Sensing Yarns after Wash Tests

This supplementary information shows results from acoustic sensing E-yarns that were wash tested. Twenty acoustic sensing yarns were wash tested. Results from two of these have already been presented in section 3.4.1 of the main paper. These two were machine washed and tumble dried within pockets attached to a T-shirt.

Results from Machine Washing and Tumble Drying Tests within Pockets Attached to a T-Shirt

Figure S1a,b show results from the wash tests of other acoustic sensing yarns that were machine washed and tumble dried within pockets attached to a T-shirt. Another yarn broke immediately after the first wash, so a graph for this is not shown.

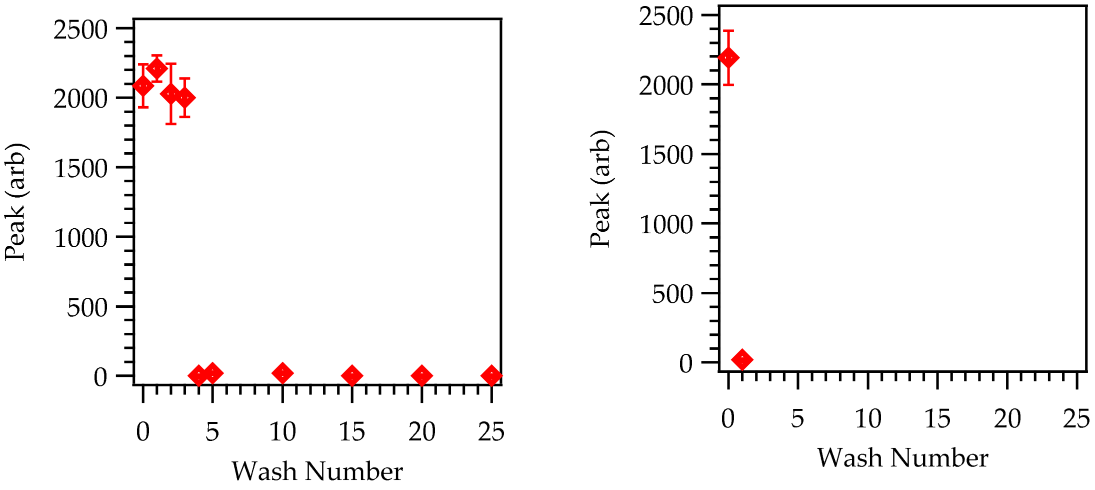

(**a**) (**b**)

Figure S1. Measurements from acoustic sensing E-yarns machine-washed and tumble dried within pockets attached to a T-shirt (**a**) An E-yarn that failed after 4 washes. (**b**) An E-yarn that stopped functioning after the first wash.

Results from Machine Washing and Tumble Drying Tests within Channels in A Woven Fabric

Figure S2 shows results from E-yarns that were placed within channels in a woven textile, then machine-washed and tumble dried. The results show that these E-yarns tended to degrade as more wash cycles were completed. Figure S2d shows results from the only E-yarn that functioned correctly after 25 washes. Note that an acoustic sensing E-yarn was deemed to be functioning correctly if, at 130 dB, it gave a response above 1665.57 arbitrary units, which was taken as the minimum output value of an acoustic sensing yarn before it was subjected to the wash (from 30 samples tested), as stated in section 2.2.4 of the paper. Other E-yarns with results shown in S2a and S2b retained partial functionality after 25 washes.

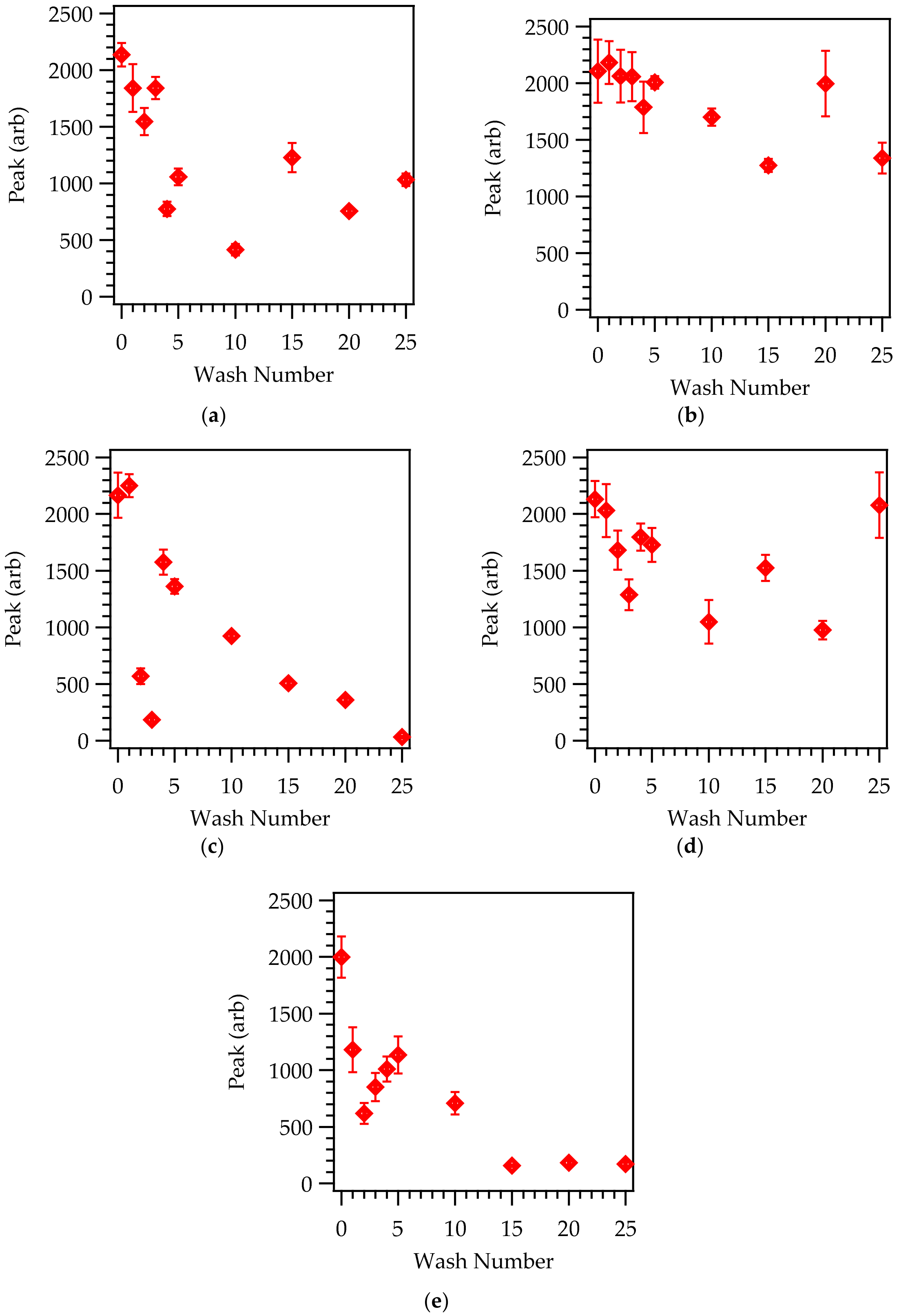

Figure 2. Acoustic E-yarns machine washed, and tumble dried within channels in a woven textile. The results show: (**a**) Some degree of reverse in the degradation of the E-yarn; (**b**) Less degradation over 25 washes; (**c**) Complete failure after 25 washes; (**d**) An E-yarn that was functioning correctly after 25 washes; (**e**) Severe degradation over 25 washes.

Results from Machine Washing and Line Drying Tests

Figures S3 and S4 show results from E-yarns that were machine washed and line dried. Figure S3 shows results from E-yarns that were placed in pockets attached to a T-shirt. These did not function correctly after wash after wash 5. The E-yarns retained some functionality, but below the 1665.57 arbitrary units that were deemed as the cut-off. The results in S3(a) show that one E-yarn retained some functionality after 25 washes.

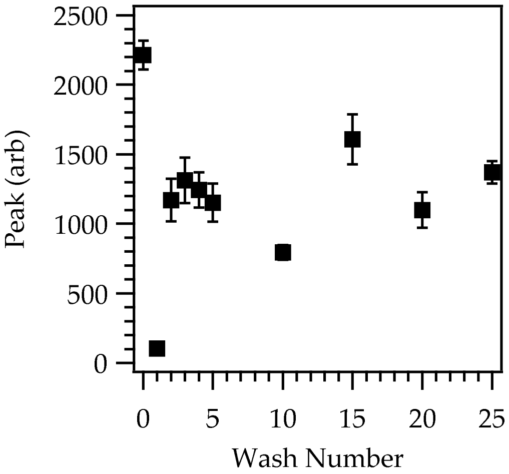

(**a**)

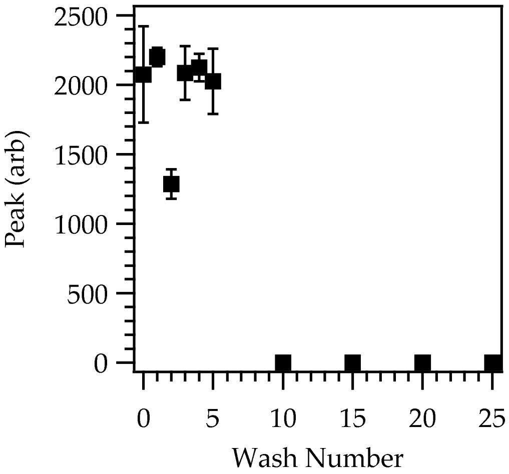

(**b**)

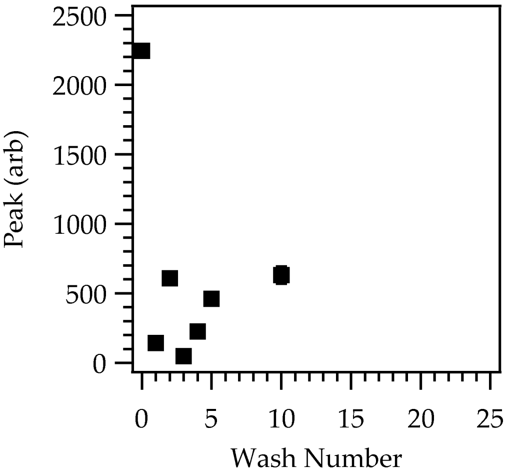

(**c**)

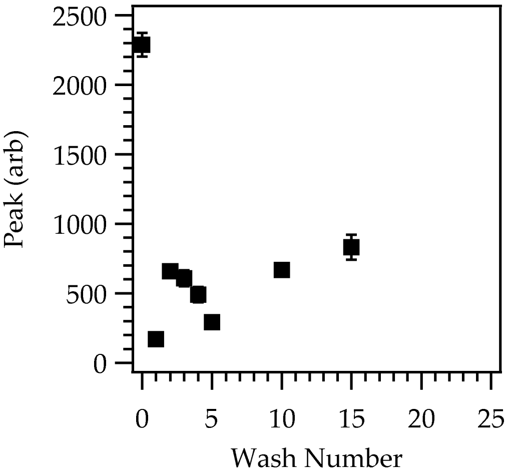

(**d**)

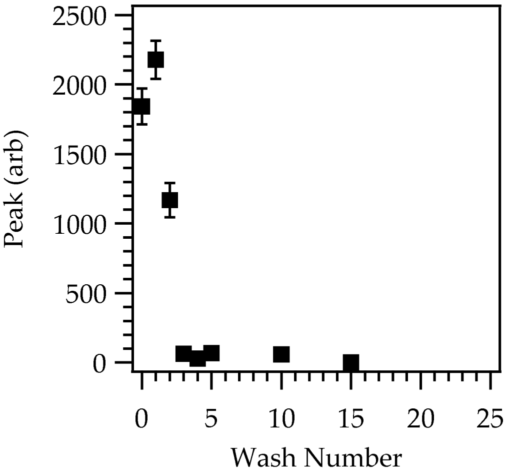

(e)

Figure S3. Acoustic E-yarns machine washed, and line dried in pockets attached to a T-shirt. The results show: (**a**) Some level of recovery after severe malfunction at the start of testing; (**b**) Complete failure after 10 washes; (**c**) Severe degradation at the start of testing, with complete failure by wash 15; (**d**) Severe degradation at the start of testing with complete failure by wash 20; (**e**) Minimal functionality after wash 3, with failure after wash 15.

Figure S4 shows results from E-yarns held within channels in a woven textile and subjected to machine washing and line drying. Three out of the five E-yarns were functioning partially after wash 25, as shown in S4 b–d.

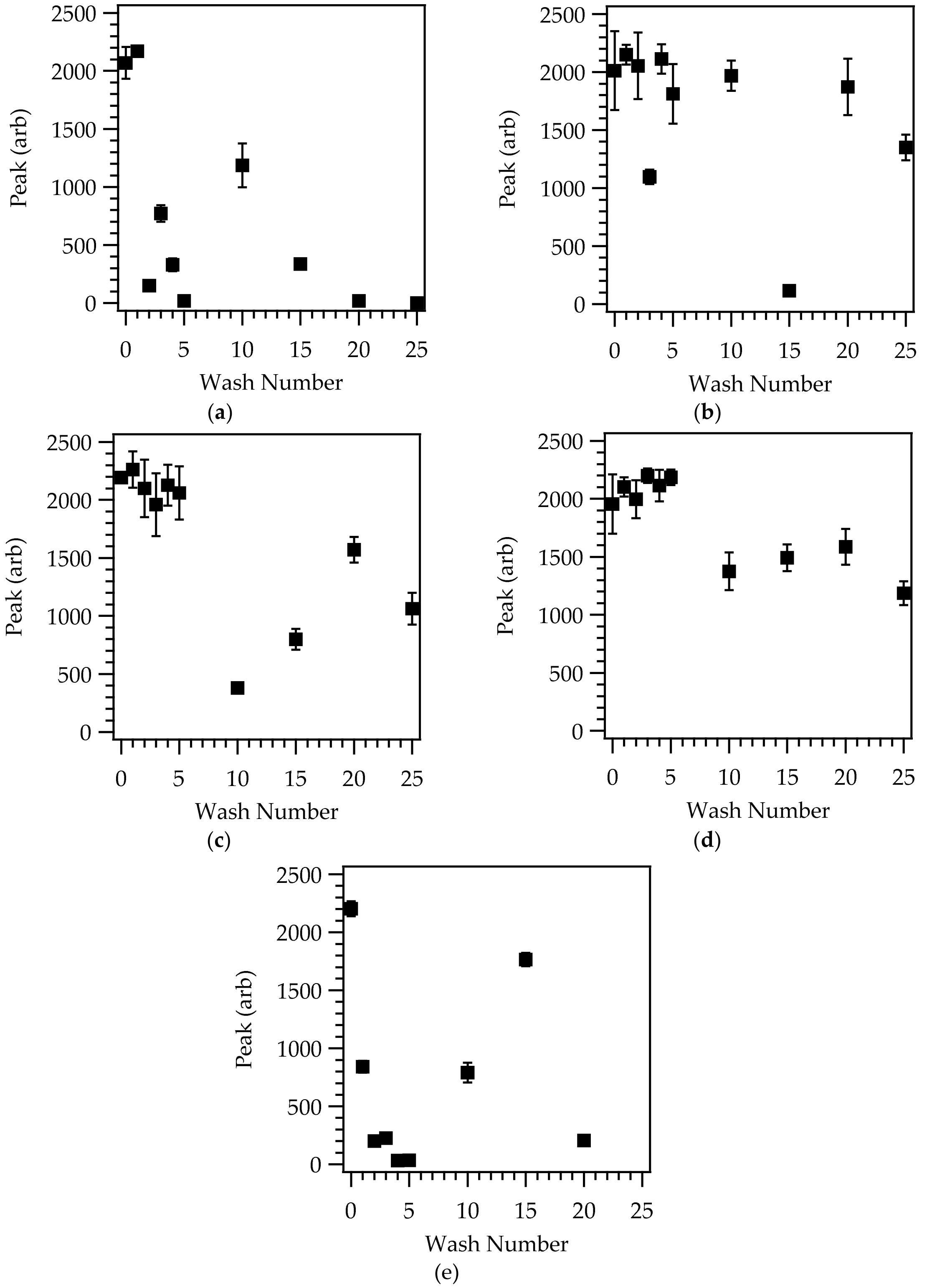

Figure 4. Acoustic E-yarns machine washed, and line dried within channels in a woven textile. Results show: (**a**) Apparent failure after wash 5 with some degree of recovery, then failure after wash 20; (**b**) Some recovery from near failure, leading to some degree functionality after 25 washes; (**c**) Some degree of functionality after 25 washes; (**d**) Some degree of functionality after 25 washes; (**e**) Complete failure after wash 25. .

© 2020 by the authors. Licensee MDPI, Basel, Switzerland. This article is an open access article distributed under the terms and conditions of the Creative Commons Attribution (CC BY) license (http://creativecommons.org/licenses/by/4.0/).
